# Supplementary material for: Treatment with Paracetamol Can Interfere with the Intradialytic Optical Estimation in Spent Dialysate of Uric Acid but Not of Indoxyl Sulfate
Source: Toxins (Basel). 2022 Sep 1;14(9):610. doi: 10.3390/toxins14090610 (PMC9502103; doi:10.3390/toxins14090610)
Supplement: Supplementary file 1 [file toxins-14-00610-s001.zip › toxins-1826802-supplementary.pdf]

Table S1. Complete list of medicines that were prescribed to the end stage kidney disease patients according to their individual medication plan.

Medicines		Specifications		
ATC code	Active ingredient(s)	Par-	Par+	Exceptional cases
A01AA01	Sodium fluoride	-	+	-
A02AA01	Magnesium carbonate	+	+	-
A02AA02	Magnesium oxide	+	-	-
A02BC01	Omeprazole	+	+	+
A02BC02	Pantoprazole	+	+	+
A03FA01	Metoclopramide	+	-	+
A06AB08	Sodium picosulfate	+	+	-
A06AD11	Lactulose	+	-	-
A06AD65	Macrogol	+	+	-
A07DA03	Loperamide	+	+	-
A10AB04	Insulin lispro	+	-	-
A10AB05	Insulin aspart	+	-	+
A10AB06	Insulin glulisine	-	+	-
A10AC01	Insulin (human)	+	+	-
A10AE04	Insulin glargine	+	-	-
A10AE05	Insulin detemir	+	-	+
A10BB12	Glimepiride	+	-	-
A10BH05	Linagliptin	+	-	-
A11CC03	Alfacalcidol	+	+	+
A11CC04	Calcitriol	+	-	+
A11CC05	Colecalciferol	+	+	+
A11EX	Vitamin B-complex	+	+	+
A12AA04	Calcium carbonate	+	+	+
A12AA07	Calcium chloride	-	-	+
A12BA01	Potassium chloride	+	-	-
B01AA03	Warfarin	+	+	-
B01AB05	Enoxaparin	+	+	+
B01AB10	Tinzaparin	+	+	+
B01AC04	Clopidogrel	+	+	-
B01AC06	Acetylsalicylic acid	+	-	-
B01AD02	Alteplase	+	+	-
B03AC02	Saccharated iron oxide	+	+	-
B03BA01	Cyanocobalamin	+	+	-
B03BB01	Folic acid	+	+	-

B03XA01	Epoetin beta	+	+	+
B03XA02	Darbepoetin alfa	+	+	-
B05AA01	Albumin	+	-	-
B05AA06	Succinylated gelatine	+	-	-
B05CB02	Sodium citrate	+	+	-
B05XA02	Sodium bicarbonate	+	+	-
C01AA05	Digoxin	+	-	-
C01BC03	Propafenone	+	-	-
C01BD01	Amiodarone	+	-	-
C01DA02	Glyceryl trinitrate	+	+	-
C01DA14	Isosorbide mononitrate	+	+	-
C02AC05	Moxonidine	+	-	+
C02CA04	Doxazosin	+	+	+
C02DB02	Hydralazine	+	-	-
C03AA03	Hydrochlorothiazide	+	-	+
C03BA08	Metolazone	+	-	-
C03CA01	Furosemide	+	+	+
C03CA02	Bumetanide	+	+	-
C03CA04	Torasemide	+	-	+
C03DA01	Spironolactone	+	+	-
C05CA53	Diosmin	-	+	-
C07AB02	Metoprolol	+	+	+
C07AB07	Bisoprolol	+	+	+
C07AB12	Nebivolol	+	-	-
C08CA01	Amlodipine	+	+	+
C08CA02	Felodipine	+	+	-
C08CA05	Nifedipine	+	-	-
C08CA08	Nitrendipine	+	-	-
C08CA13	Lercanidipine	+	+	-
C08DB01	Diltiazem	+	-	-
C09AA02	Enalapril	+	-	-
C09AA04	Perindopril	+	+	-
C09AA05	Ramipril	+	-	-
C09CA01	Losartan	+	+	-
C09CA06	Candesartan	+	+	-
C09CA07	Telmisartan	+	-	-
C09CA08	Olmesartan medoxomil	+	-	+
C10AA01	Simvastatin	+	+	-
C10AA05	Atrovastatin	+	+	-

C10AA07	Rosuvastatin	+	-	-
C10AX09	Ezetimibe	-	+	-
D01AC08	Ketoconazole	+	-	-
D07AC01	Betamethasone	+	+	-
D07AC13	Mometasone	+	-	-
D07XA01	Hydrocortisone	+	+	-
D11AX18	Diclofenac	-	+	-
G01AF01	Metronidazole	+	-	-
G03AC09	Desogestrel	+	-	-
G03CA03	Estradiol	-	+	-
G04BE03	Sildenafil	+	-	-
G04CA01	Alfuzosin	+	-	-
G04CA02	Tamsulosin	+	-	-
G04CB01	Finasteride	-	+	-
H02AB01	Betamethasone	+	+	-
H02AB04	Methylprednisolone	+	-	+
H02AB06	Prednisolone	+	+	+
H03AA01	Levothyroxine sodium	+	-	+
H05BX01	Cinacalcet	+	-	-
H05BX02	Paricalcitol	+	-	-
J01CA04	Amoxicillin	-	+	-
J01CF04	Oxacillin	+	-	-
J01CF05	Flucloxacillin	-	-	+
J01CR01	Ampicillin and beta-lactamase inhibitor	-	-	+
J01DD01	Cefotaxime	-	+	-
J01FA09	Clarithromycin	+	-	-
J01FF01	Clindamycin	-	+	-
J05AB11	Valaciclovir	-	-	+
L02BA01	Tamoxifen	+	-	-
L02BB03	Bicalutamide	+	-	-
L04AA06	Mycophenolic acid	+	-	-
L04AA10	Sirolimus	+	-	-
L04AA18	Everolimus	+	-	-
L04AD01	Ciclosporin	-	+	-
L04AD02	Tacrolimus	+	-	-
M01AE03	Ketoprofen	+	-	-
M04AA01	Allopurinol	+	+	+
M04AA03	Febuxostat	+	-	-
N01BB02	Lidocaine	+	+	-

N02AA05	Oxycodone	+	+	-
N02AE01	Buprenorphine	-	+	-
N02AX02	Tramadol	-	+	-
N02BA01	Acetylsalicylic acid	+	+	-
N02BE01	Paracetamol	-	+	+
N03AX12	Gabapentin	+	+	-
N03AX16	Pregabalin	+	-	-
N05AH03	Olanzapine	+	-	-
N05BA04	Oxazepam	+	+	-
N05BA12	Alprazolam	+	-	-
N05BB01	Hydroxyzine	+	+	-
N05CD02	Nitrazepam	-	+	-
N05CD03	Flunitrazepam	+	+	-
N05CF01	Zopiclone	+	+	-
N05CF02	Zolpidem	+	-	-
N06AA09	Amitriptyline	+	+	-
N06AB05	Paroxetine	-	+	-
N06AB06	Sertraline	+	-	-
N06AX05	Trazodone	+	-	-
N06AX21	Duloxetine	+	-	-
R01AB05	Ephedrine	-	+	-
R01AD09	Mometasone	+	-	-
R03AC02	Salbutamol	+	+	-
R03AC04	Fenoterol	+	-	-
R03AC12	Salmeterol	+	-	-
R03AC13	Formoterol	+	+	-
R03AC18	Indacaterol	+	-	-
R03BA01	Beclometasone	-	+	-
R03BA02	Budesonide	+	+	-
R03BA05	Fluticasone	+	-	-
R03BB01	Ipratropium bromide	+	+	-
R03BB04	Tiotropium bromide	+	+	-
R03BB06	Glycopyrronium bromide	+	-	-
R03DC03	Montelukast	+	-	-
R05CB01	Acetylcysteine	+	+	-
R05CB02	Bromhexine	-	+	-
R06AA04	Clemastine	+	+	-
R06AD02	Promethazine	-	-	+
R06AX13	Loratadine	+	+	-

R06AX27	Desloratadine	+	-	-
S01EC03	Dorzolamide	+	-	-
S01ED01	Timolol	+	+	-
S01EE04	Travoprost	+	-	-
S01GX01	Cromoglicic acid	+	+	-
S01XA20	Hydroxypropyl methylcellulose	+	-	-
V03AE01	Polystyrene sulfonate	+	+	+
V03AE02	Sevelamer	+	+	+
V03AE03	Lanthanum carbonate	+	+	-
V03AE07	Calcium acetate	+	+	-

ATC - The Anatomical Therapeutic Chemical code. All patients received medicines according to their prescribed medication plan, including UV (200–400 nm) absorbing drugs. The medicines that were taken during week prior the dialysis or during the dialysis procedure are included. Dialysis treatments were divided into two groups based on UV absorbing drugs administration. In total, 137 dialysis treatments were included into the Par- group where Par was not prescribed and any of the prescriptions included UV absorbing medications did not exceeded in total 500 mg per day. Altogether, 30 treatments were included into the Par+ group where additionally Par was prescribed to patients as described in Table 2 (daily doses >500 mg). Exc: Including data of 6 exceptional treatments sessions of 3 patients, who had received ampicillin, flucloxacillin (together with Par) or valaciclovir in high dosage that were excluded from the analysis of paracetamol influence.
